# Shaving the Beard

**Published:** 1877-12

**Authors:** 


### SHAVING THE BEARD.

The more I reflect upon the mysteries of neurology and animal chemistry, the more confident I am that, while we are the least suspecting it, trifling errors in our daily life are producing important effects upon our corporeal systems; and I declare it as my deliberate conviction, that the habit, which may almost be styled American, of using the razor upon the face, is sufficient to cause a large proportion of the lamentable evils which affect the human race in this country.

It appears by experiment that the beard, if shaved, grows four to five times faster than if unshorn. In this calculation, an item is omitted which it is difficult to estimate, *i. e.*, the stimulus given the beard, by the first application of the razor in adolescence, the experiments being made upon beards after they have acquired an unnaturally rapid growth. The effect of this early stimulus may be fairly counted at double the natural growth; then reckoning the difference in size and weight of the fibre, which is treble, and we find the frightful truth to be, that we raise thirty times the natural quantity of beard! Thus it is evident that the true beard is exhausted at a very early age, after which the system is forced to supply a substitute. Now nature will not submit with impunity to extraordinary demands upon her vigor, and that which requires her to produce in a lifetime thirty times as much beard as she was first inclined to, must certainly be considered as such. She is fatigued in proportion to the effort, let the particular kind be what it may; although her recuperative powers are great, she insists upon having repose, even when working at a rate chosen by herself. If that repose is denied her, she takes her revenge by breaking down the mechanism.

